# Process Optimization, Morphology, Structure, and Adhesive Strength of Electrodeposited Ni–Fe–Graphene Composite Coating on the 7075 Aluminum Alloy

**DOI:** 10.3390/ma16176062

**Published:** 2023-09-04

**Authors:** Na Li, Lan Zhang, Huizhong Ma, Qiao Li, Xingke Sun

**Affiliations:** 1 School of Mechanics and Safety Engineering, Zhengzhou University, Zhengzhou 450001, China; iehzma@zzu.edu.cn (H.M.); 15838468176@163.com (X.S.); 2 College of Computer and Communication Engineering, Zhengzhou University of Light Industry, Zhengzhou 450000, China; liqiaoself@126.com

**Keywords:** electrodeposition, Ni–Fe–graphene, process optimization, hardness, friction coefficient

## Abstract

The process parameters of electrodeposited Ni–Fe–graphene composite coating on the 7075 aluminum alloy were optimized by the orthogonal experiment. The optimized process parameters were determined as follows: graphene concentration of 1 g L^−1^, current density of 9 A dm^−2^, agitation speed of 250 r min^−1^, and temperature of 60 °C, on the basis of hardness and friction coefficient. The Ni–Fe–graphene composite coating shows an increment of 393.0% in hardness and a decrement of 55.9% in friction coefficient in comparison with 7075 aluminum alloy substrate. The Ni–Fe–graphene composite coating binds tightly to 7075 aluminum alloy with adhesion strength of higher than 6.895 MPa. These make contributions to provide effective protection for aluminum alloys. Surface morphology and corrosion morphology, as well as morphology of the side bound to the substrate, were characterized. The scattered asperities on the surface were proven to be graphene nanoplatelets being wrapped by Ni–Fe, which comprehensively reveals the formation of asperities.

## 1. Introduction

Aluminum and its alloys are extensively applied to aeronautical and aerospace, automobile, and vessel domains because of their merits, including high specific strength, superior electrical and thermal conductivity, and low cost. However, as the demands for materials possessing low friction, high strength, and lightweight continue to escalate, the weak spots of low surface hardness and poor wear resistance for aluminum alloys are not only increasingly prominent, but also limit their application range, and even lead to huge economic loss due to wear failure. Therefore, it is very crucial for aluminum and its alloys to improve surface hardness and reduce friction and wear, utilizing surface modification and reinforcement methods.

Electrodeposited Ni–Fe alloy coating has been extensively applied to electronic sensors and micromechanical systems due to its merits such as homogenous structure, eminent magnetic properties [1], corrosion resistance [2] and mechanical properties [3]. Moreover, less nickel not only saves energy but can also decrease production cost. The change of Fe content in the coating or the addition of particles could endow Ni–Fe alloy coating with different mechanical properties to meet different needs [4,5,6,7]. Related research has demonstrated that the addition of SiC, TiO_2_, TiN, WC, Cr_2_O_3_, AlN, and MWCNT is beneficial to increase hardness and decrease friction coefficient. The properties of different Ni–Fe composite coatings are listed in Table 1. Up to now, reports on the wear behavior of electrodeposited Ni–Fe composite coatings have been very limited.

Graphene, an atom-thick of carbon, has extraordinary mechanical property, including elasticity modulus of 0.5~1.0 Tpa [17], tensile strength of 150~180 GPa [18], and fracture strength of 125 GPa [19]. Simultaneously, it has shown great promise as a high-performance solid lubricant [20] or liquid lubricant additive [21,22] due to its ultrathin configuration, two-dimensional structure, high load carrying capacity, and extreme strength [23,24,25,26,27]. Electrodeposited metal matrix composite coatings being reinforced by graphene have demonstrated that the insertion of graphene is beneficial for significant improvement in hardness [28], strength [29], corrosion resistance [30], wear resistance, and antifriction [27]. Surbramanya et al. [31] developed a Ni–Fe–graphene composite electrode by embedding graphene into the Ni–Fe matrix, and the hydrogen production of Ni–Fe–graphene composite electrode was three times higher than Ni–Fe alloy. Our previous research observed excellent mechanical properties [32] and wear resistance [33] of electrodeposited Ni–Fe–graphene coating, which provide effective protection for aluminum alloy, 45# steel, and stainless steel.

Up to now, there have been quite limited reports about electrodeposited Ni–Fe–graphene composite coating on the 7075 aluminum alloy and process optimization. In this work, the process parameters of Ni–Fe–graphene composite coatings are optimized by orthogonal design. The morphology, structure, and adhesive strength are investigated as well. 

## 2. Experimental

### 2.1. Pretreatment of Substrate

An aluminum alloy (#7075) plate with size of 25 × 25 × 3 mm was used as a cathode for coatings deposition, and a pure nickel plate was used as an anode. The cathode and anode were positioned parallel at an angle of 45 degrees with the plating solution level. The aluminum alloy substrates were polished with 600# emery paper to remove the surface scratches and obtain a uniform surface, and then were sandblasted using 280 mesh glass beads under pressure of about 0.15 MPa to eliminate the oxidation film and increase the coating’s adhesion. Subsequently, the samples were cleaned ultrasonically and chemically treated for a few minutes in 3%NaOH and 3%H_2_SO_4_ to further remove impurities and then were ultrasonic-cleaned in deionized water again.

### 2.2. Preparation of Ni–Fe–Graphene Composite Coating

In this study, graphene concentration, current density, agitation speed, and temperature were sieved by the orthogonal designing method, and the orthogonal test table of L16 (4^4^) is shown in Table 2.

The electrodeposition of the Ni–Fe–graphene composite coating was carried out under the direct current conditions from the alkaline Ni–Fe alloy bath solution in Table 3. The pH of bath solution was kept at 8.5 and the electrodeposition time was kept at 2 h. 

**Figure 1:**
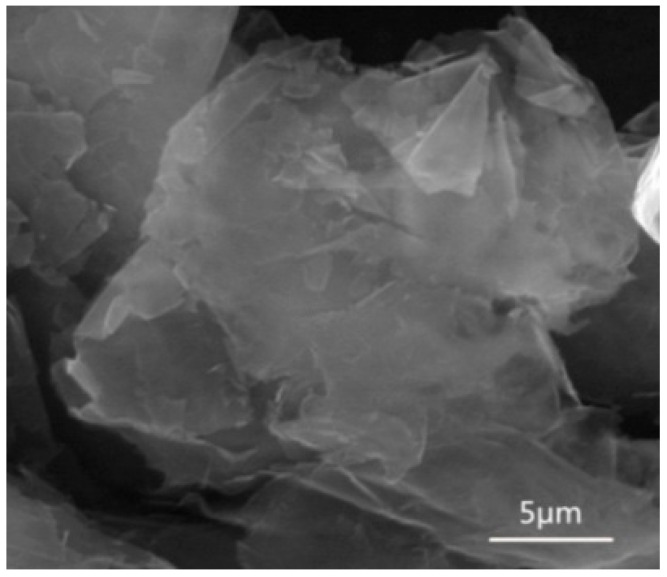
SEM image of graphene nanoplatelets.

All chemicals were of analytical grade. Graphene nanoplatelets, supplied by Chengdu Organic Chemicals Co., Ltd., Chinese Academy of Sciences (Chengdu, China), were about 4~20 nm in thickness and 5~10 μm in diameter. An SEM image of the graphene nanoplatelets is shown in Figure 1.

Prior to electrodeposition, in order to ensure the dispersion of graphene in the electrolyte, the bath solution was stirred for 30 min using an ultrasonic emulsification distributor and subsequently stirred mechanically for about 1 h. To maintain the suspension of graphene nanoplatelets in the electrolyte during the deposition process, mechanical agitation was kept constant. After the deposition, samples were cleaned to remove the residual solution, then dried, and then measured. 

### 2.3. Characterization and Measurement of Coatings

The adhesion strength between the Ni–Fe–graphene composite coating and the aluminum alloy substrate was evaluated by the Elcometer model 106 adhesion tester (Elcometer Instruments Ltd., Manchester, UK). It employs the pull-off method to measure the lift-off force required to pull a small area of coating away from the base material. First, the surface of the dolly and Ni–Fe–graphene composite coatings were roughened with 1200 mesh abrasive paper, then degreased in absolute ethyl alcohol by ultrasonic cleaning. E-44 epoxy was mixed with low-molecular-weight 650 polyamide resin in a mass ratio of 1:1. The mixture, namely, adhesive, was applied as an even film to the surface of dolly. The dolly was placed onto the surface of the Ni–Fe–graphene composite coating and pressure was applied to squeeze out the excess adhesive being removed. Then, we placed them in the drying oven with a temperature of 60 °C for 3 h. After curing, the support ring was placed over the dolly, ensuring it lay flat. The pull-off force was applied to the test dolly until the dolly was removed from the surface. The pull-off force was recorded by means of a dragging indicator on an engraved scale. Three samples were prepared under the same process parameters and were measured.

The morphology and element composition were characterized using a scanning electron microscope (SEM, FEI Quanta 200, FEI company, Hillsboro, OR, USA) equipped with energy-dispersive spectroscopy (EDS). 

The Vickers hardness of the Ni–Fe–graphene composite coatings was examined using a microhardness tester (HXD-1000, Shanghai Taiming Optical Instrument Co., Ltd., Shanghai, China) with a load of 100 g applied for 10 s. Five data points were averaged for each sample. All coatings and substrate suffered from friction testing on a ball-on-disk tribometer (Model MS-T3000; Lanzhou Institute of Chemical Physics, Chinese Academy of Sciences, Lanzhou, China) under dry air environment at room temperature. The counterpart ball for wear test was a GCr15 steel ball with diameter of 3 mm. The test was carried out at the sliding velocity of 0.1 m s^−1^, keeping the applied normal load (F, N) of 1.0 N for 30 min. The test was repeated three times for each material under the same test conditions. 

## 3. Results and Discussion

### 3.1. Process Optimization

The process parameters of the Ni–Fe–graphene composite coatings were optimized according to the hardness and friction coefficient (COF). The orthogonal test results are shown in Table 4. According to hardness, the optimized process parameters are graphene concentration of 1 g L^−1^, current density of 9 A dm^−2^, agitation speed of 250 r min^−1^, and temperature of 60 °C, corresponding to experiment No. 4 in Table 4. The hardness and friction coefficient of the Ni–Fe–graphene composite coating prepared under these parameters is 944.9 HV and 0.2221, respectively. However, according to friction coefficient, the optimized process parameters are graphene concentration of 3 g L^−1^, current density of 5 A dm^−2^, agitation speed of 100 r min^−1^, and temperature of 60 °C, corresponding to experiment No. 6 in Table 4. The hardness and friction coefficient of the Ni–Fe–graphene composite coating prepared under these parameters is 621.5 HV and 0.1567, respectively. The optimized process parameters for the different indexes show a discrepancy. Therefore, we needed to evaluate the properties of the Ni–Fe–graphene prepared under the optimized parameters for different indexes comprehensively, and the evaluation results are shown in Table 5. The results show that the comprehensive properties of the Ni–Fe–graphene for experiment No. 6 are even worse than experiment No. 4. Hence, the optimized parameters are consistent with experiment No. 4 in Table 4. 

In addition to the process parameters’ optimization by analyzing orthogonal test results visually, range analysis was conducted on the basis of orthogonal test results, and the range analysis results are shown in Table 6 (according to hardness) and Table 7 (according to COF). 

A is representative of hardness, and A_1_, A_2_, A_3_, and A_4_ are responding to the average hardness of the same level under different factors (graphene concentration, current density, agitation speed, and temperature). B is representative of COF, and B_1_, B_2_, B_3_, and B_4_ are responding to the average COF of the same level under different factors (graphene concentration, current density, agitation speed, and temperature). R_i_ is representative of hardness range and R_j_ is representative of COF range. The influence degree of different factors on properties is confirmed and the higher the value of the range, the greater the degree of influence. 

The influence degree on the hardness of the Ni–Fe–graphene composite coating of different factors, from the highest to the lowest, is graphene concentration, current density, agitation speed, and temperature. The optimized parameters according to the average hardness under the different levels are responding to experiment No. 4, which agrees with the optimized parameters by analyzing orthogonal test results visually. 

However, the influence degree on the COF of the Ni–Fe–graphene composite coating of different factors, from the highest to the lowest, is current density, agitation speed, graphene concentration, and temperature. The optimized parameters according to the average COF under the different levels are graphene concentration of 3 g L^−1^, current density of 9 A dm^−2^, agitation speed of 100 r min^−1^, and temperature of 40 °C, which is different from any experiment in Table 4 and experiment No. 4. Therefore, the Ni–Fe–graphene composite coating was prepared again under the process parameters of experiment No. 4 (No. 17) and the optimized parameters by COF range analysis (No. 18), respectively. The results are shown in Table 8. 

As a result, the optimized process parameters are graphene concentration of 1 g L^−1^, current density of 9 A dm^−2^, agitation speed of 250 r min^−1^, and temperature of 60 °C. The hardness and friction coefficients of the Ni–Fe–graphene composite coating prepared under these parameters are 912.6 HV and 0.1990, respectively. In comparison with 7075 aluminum alloy substrate, the Ni–Fe–graphene composite coating shows an increment of 393.0% in hardness and a decrement of 55.9% in friction coefficient.

### 3.2. Morphology and Composition

**Figure 2:**
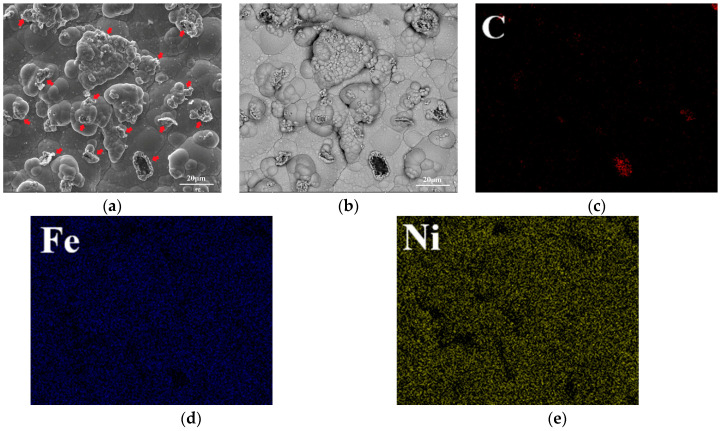
Ni–Fe–graphene composite coatings: (**a**) SEM image; (**b**) backscatter electron image; (**c**) C element; (**d**) Fe element; (**e**) Ni element.

The surface morphology and element distribution of Ni–Fe–graphene composite coatings are shown in Figure 2. A large number of asperities are distributed on the compact surface. These asperities are graphene being wrapped by Ni–Fe alloy completely or incompletely being marked by red arrows (Figure 2a), which is verified further by backscatter electron image (Figure 2b) and surface energy spectrum analysis in Figure 2c–e corresponding to Figure 2b.

The element compositions of the Ni–Fe–graphene composite coatings from EDS analysis are shown in Table 9. It was found that the C content in the Ni–Fe–graphene composite coating was higher than that in the Ni–Fe alloy owing to the incorporation of GNPs.

**Figure 3:**
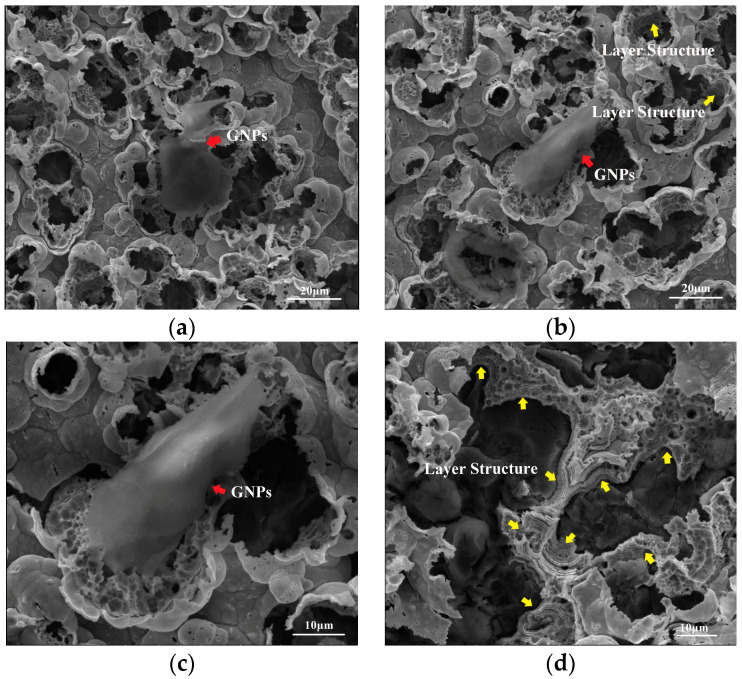
Corrosion surface morphology of Ni–Fe–graphene (**a**,**b**), (**c**) enlarged image in (**b**), (**d**) layer structure in the corrosion surface.

In order to further prove the asperities being scattered on the surface, the Ni–Fe–graphene composite coatings were corroded in 30 vol% HNO_3_. The corrosion morphology is presented in Figure 3. After the surface of asperities is corroded, holes with different sizes appear and the incorporated graphene is exposed, as shown by red arrows. Simultaneously, it presents an obvious layer structure, indicated by yellow arrows, especially in Figure 3d, which is caused by incorporation of graphene.

Ni^2+^ and Fe^2+^ ions are reduced preferentially on the GNPs after GNPs are deposited on the cathode, which realize the incorporation of GNPs into the composite coating. Due to excellent electrical conductivity and the large specific surface area of graphene, the deposition rate of metal ions on the GNP surface exceeds that on the reduced Ni–Fe alloy, which modifies the surface morphology and induces the formation of asperities by offering more active sites for metal atoms [32,34,35]. When GNPs are decorated by Ni–Fe alloy, Ni^2+^ and Fe^2+^ ions continue to be reduced on the surface as electrodeposition proceeds, which generates a layer structure on the wall of holes. The results reveal the formation of asperities and electrodeposited graphene composite coatings. 

**Figure 4:**
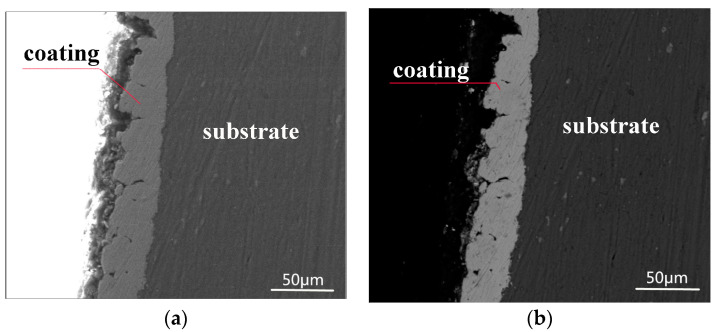
Cross-sectional morphology of Ni–Fe–graphene (**a**,**b**).

Figure 4 depicts the cross-sectional morphology of the Ni–Fe–graphene composite coating. The Ni–Fe–graphene is well bonded to the aluminum alloy substrate and it is uniform and compact. The thickness is approximately 30.0 μm. The adhesion strength between the composite coatings and substrate was examined by an F106 adhesion tester, which employs the pull-off method to measure the force that pulls a small area of coating away from the substrate. 

**Figure 5:**
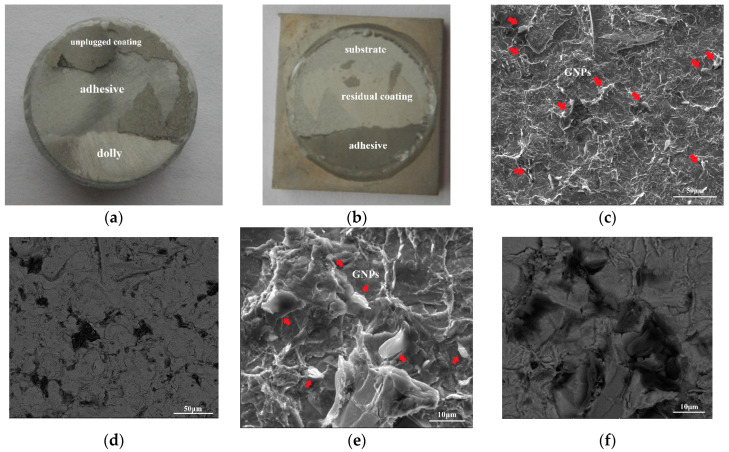
Adhesion strength test images of (**a**) dolly; (**b**) corresponding to substrate; (**c**,**e**) SEM morphology of Ni–Fe–graphene close to substrate; (**d**,**f**) backscatter electron image corresponding to (**c**,**e**).

Dollies were separated from between the Ni–Fe–graphene composite coating and the substrate. The separated area is equivalent to 30%, as shown in Figure 5a,b. The average value is 6.895 ± 0.159 MPa, which indicates that the adhesion strength between composite coatings and substrate is higher than 6.895 ± 0.159 MPa. Simultaneously, the morphology of the Ni–Fe–graphene close to the substrate was characterized, as shown in Figure 5c–f, after it was separated from the substrate. There are many GNPs dispersed in the composite coating, marked by red arrows, which demonstrates that GNPs are attached to the substrate surface at the initial deposition stage and then are embedded into the composite coating as the deposition process continues. 

## 4. Conclusions

A Ni–Fe–graphene composite coating was prepared on the 7075 aluminum alloy using the electrodeposition technique, and the process parameters were optimized by the orthogonal experiment. The optimum process parameters are graphene concentration of 1 g L^−1^, current density of 9 A dm^−2^, agitation speed of 250 r min^−1^, and temperature of 60 °C. The Ni–Fe–graphene composite coating shows an increment of 393.0% in hardness and a decrement of 55.9% in friction coefficient in comparison with the 7075 aluminum alloy substrate. The Ni–Fe–graphene composite coating binds tightly to the 7075 aluminum alloy and the adhesion strength is higher than 6.895 MPa. The incorporation of graphene modifies the surface morphology and induces the formation of asperities. These asperities are graphene being wrapped completely or incompletely, with Ni–Fe alloy being verified by surface morphology, composition, and corrosion surface morphology. Simultaneously, layered structures in the corrosion holes clarify the formation of electrodeposited graphene reinforced metal matrix composite coating.

## Figures and Tables

**Table 1:** Hardness and friction coefficient of electrodeposited Ni–Fe composite coatings.

Type of Coatings	Hardness (HV)	Friction Coefficient	Reference
Ni–Fe/SiC	710	—	[8]
	—	0.6	[9]
Ni–Fe–TiO_2_	638	0.52	[10]
	526	—	[11]
	647	—	[12]
Ni–Fe–TiN	660	—	[13]
Ni–Fe–WC	569.2	0.727	[14]
Ni–Fe–Cr_2_O_3_	565	0.72	[15]
Ni–Fe–AlN	560	—	[16]
Ni–Fe–MWCNT	—	0.38	[9]

**Table 2:** Orthogonal test table of L16 (4^4^).

	Factors	Graphene Concentration (g/L)	Current Density (A dm^−2^)	Agitation Speed (r min^−1^)	Temperature (°C)
Levels					
1		1	3	100	30
2		1	5	150	40
3		1	7	200	50
4		1	9	250	60
5		3	3	150	50
6		3	5	100	60
7		3	7	250	30
8		3	9	200	40
9		5	3	200	60
10		5	5	250	50
11		5	7	100	40
12		5	9	150	30
13		7	3	250	40
14		7	5	200	30
15		7	7	150	60
16		7	9	100	50

**Table 3:** Bath composition.

Composition	Content (g L^−1^)
NiSO_4_·6H_2_O	16.8
FeSO_4_·7H_2_O	11.1
H_3_BO_3_	15
C_6_H_8_O_6_	1
Na_2_SO_4_	10
C_6_H_5_O_7_(NH_4_)_3_	45
C_10_H_16_N_2_O_8_	2.4

**Table 4:** Orthogonal test results.

	Factors	Graphene Concentration (g L^−1^)	Current Density (A dm^−2^)	Agitation Speed (r min^−1^)	Temperature (°C)	Hardness (HV)	COF
No.							
1		1	3	100	30	534.8 (±14.50)	0.2334 (±0.018)
2		1	5	150	40	788.4 (±25.55)	0.2118 (±0.015)
3		1	7	200	50	772.2 (±25.78)	0.2146 (±0.016)
4		1	9	250	60	944.9 (±35.66)	0.2221 (±0.012)
5		3	3	150	50	283.6 (±28.36)	0.3080 (±0.010)
6		3	5	100	60	621.5 (±18.33)	0.1567 (±0.015)
7		3	7	250	30	673.4 (±26.25)	0.2167 (±0.009)
8		3	9	200	40	542.9 (±24.10)	0.1687 (±0.009)
9		5	3	200	60	191.8 (±4.22)	0.3549 (±0.016)
10		5	5	250	50	422.5 (±39.00)	0.2401 (±0.019)
11		5	7	100	40	483.7 (±30.09)	0.1856 (±0.017)
12		5	9	150	30	513.0 (±26.36)	0.1668 (±0.012)
13		7	3	250	40	271.4 (±28.36)	0.2777 (±0.004)
14		7	5	200	30	551.0 (±21.67)	0.2397 (±0.012)
15		7	7	150	60	536.4 (±24.26)	0.2651 (±0.029)
16		7	9	100	50	590.7 (±23.83)	0.2326 (±0.018)

**Table 5:** Comprehensive evaluation result for Ni–Fe–graphene.

	Index	Hardness (HV)	COF
No.			
4		944.9	0.2221
6		621.5	0.1567
Variation		34.2% decrease (harmful)	29.4% decrease (beneficial)

**Table 6:** Range analysis results according to hardness.

	Factors	Graphene Concentration (g L^−1^)	Current Density (A dm^−2^)	Agitation Speed (r min^−1^)	Temperature (°C)
Level					
A_1_		760.1	320.4	557.7	568.1
A_2_		530.3	595.9	530.4	521.6
A_3_		402.8	616.5	514.5	517.3
A_4_		487.4	647.9	578.1	573.7
R_i_		357.3	327.5	63.6	56.4

**Table 7:** Range analysis results according to COF.

	Factors	Graphene Concentration (g L^−1^)	Current Density (A dm^−2^)	Agitation Speed (r min^−1^)	Temperature (°C)
Level					
B_1_		0.2205	0.2935	0.2021	0.2142
B_2_		0.2125	0.2121	0.2379	0.2110
B_3_		0.2369	0.2205	0.2445	0.2488
B_4_		0.2538	0.1976	0.2392	0.2497
R_j_		0.0413	0.0959	0.0424	0.0387

**Table 8:** Properties of Ni–Fe–graphene prepared under different optimized process parameters.

	Factor	Graphene Concentration (g L^−1^)	Current Density (A dm^−2^)	Agitation Speed (r min^−1^)	Temperature (°C)	Hardness (HV)	COF
No.							
17		1	9	250	60	912.6	0.1990
18		3	9	100	40	592.4	0.2467

**Table 9:** Element composition of Ni–Fe–graphene.

Composition	Fe (wt%)	Ni (wt%)	C (wt%)	O (wt%)
Ni–Fe	40.21	56.43	2.32	1.03
Ni–Fe–graphene	41.62	52.08	5.33	0.97

## Data Availability

Data is unavailable due to privacy.
